# Multidimensional Compressed Sensing MRI Using Tensor Decomposition-Based Sparsifying Transform

**DOI:** 10.1371/journal.pone.0098441

**Published:** 2014-06-05

**Authors:** Yeyang Yu, Jin Jin, Feng Liu, Stuart Crozier

**Affiliations:** School of Information Technology and Electrical Engineering, the University of Queensland, St Lucia, Queensland, Australia; Institute of Psychology, Chinese Academy of Sciences, China

## Abstract

Compressed Sensing (CS) has been applied in dynamic Magnetic Resonance Imaging (MRI) to accelerate the data acquisition without noticeably degrading the spatial-temporal resolution. A suitable sparsity basis is one of the key components to successful CS applications. Conventionally, a multidimensional dataset in dynamic MRI is treated as a series of two-dimensional matrices, and then various matrix/vector transforms are used to explore the image sparsity. Traditional methods typically sparsify the spatial and temporal information independently. In this work, we propose a novel concept of tensor sparsity for the application of CS in dynamic MRI, and present the Higher-order Singular Value Decomposition (HOSVD) as a practical example. Applications presented in the three- and four-dimensional MRI data demonstrate that HOSVD simultaneously exploited the correlations within spatial and temporal dimensions. Validations based on cardiac datasets indicate that the proposed method achieved comparable reconstruction accuracy with the low-rank matrix recovery methods and, outperformed the conventional sparse recovery methods.

## Introduction

Dynamic MRI (dMRI) plays a vital role in many clinical applications, such as cardiac, perfusion and functional brain imaging. In these applications, high spatial-temporal resolution is desired to reveal anatomical details and physiological dynamics. Conventionally, the data is acquired in chronological order adhering to Nyquist sampling theorem, making MRI a relatively slow imaging modality. Routine methods speed up the MRI acquisition using a combination of fast gradient and Radio Frequency (RF) pulsing with full *k-*space sampling [1], [2]. However, owing to hardware and physiological constraints, achieving high spatiotemporal resolutions with hardware intensive sequences is technologically challenging.

Instead of increasing the data sampling rate, various approaches, including Compressed Sensing (CS) [3], have attempted to reconstruct full field-of-view (FOV) images from sub-Nyquist acquisitions. CS has been recently applied to MRI to accelerate the data collecting process. The pioneering work of applying CS to MRI to accelerate the data collecting process can be found in [4], [5]. CS states that a faithful reconstruction of the signal is achievable with a sampling rate far lower than the Nyquist limit, provided that the signal has a sparse representation in some transform basis (called the ‘sparsity basis’), which must be incoherent with the sensing matrix (i.e., Fourier transform in MRI) [3], [6]. In static MRI and dMRI, the incoherence between the sensing basis and the sparsity basis can be achieved by randomly acquiring data in the *k-*space or *k*-*t* space [3], [7]. Both the predefined sparsity bases [8] and the data-dependent (also called data-derived) transforms [9], [10] have provided successful reconstructions in static MRI applications.

CS has also been applied to dMRI, where the data sets are naturally higher-order tensors (for instance, a *third*-order tensor for a cine MRI and a *fourth*-order tensor for a volume dMRI). Conventionally, 2D/1D sparsity bases were used to account for the spatial and temporal sparsity. When the method *k-t* SPARSE [4] was applied to the cine cardiac data, the 2D wavelet transform was first applied in the spatial domain, followed by the 1D Fourier transform along the temporal dimension. The non-linear conjugate gradient algorithm [11] was then used to reconstruct the sparsity coefficients. This is a practical and straightforward extension of the SPARSE MRI [8] as used in the static scenario. However, using 2D wavelet transforms may generate smooth/blurry reconstructions at the image boundaries. Alternatively, the *k-t* FOCUSS method [12], [13] applied different transforms to sparsify diverse MRI signals and explored the temporal sparsity by employing Principle Component Analysis and Fourier transform for the aperiodic and periodic/pseudo-periodic data, respectively. Then the recursively weighted minimum norm reconstruction algorithm (called ‘FOCUSS’) [14], was used to reconstruct the sparsity coefficients. Also using the FOCUSS algorithm, the *k-t* ISD [16] improved the CS reconstruction by exploiting the support information from the *x-f* space. Recent methods studied the anatomical structures or features [17], [18] to further improve the reconstruction. Extending the application of sparsity, theoretical works [19]–[25] have investigated the low-rank matrix completion/recovery for more efficient signal recovery. The applications of the low-rank matrix structure have demonstrated merits in exploring the spatial-temporal signal redundancy in dMRI. For example, the methods described in [26]–[34] used sparse sampling schemes for data acquisition, and then generated basis functions for low-rank regularisation or to model the dMRI signals. The function bases in methods [26]–[28] were tailored from the training data of the objects, they were more capable of capturing the correlations among the dynamic image series. The quality of the reconstructions achieved by these methods, however, relied heavily on the quality of the training data. Some other methods [29]–[34] used the combination of sparse sampling and low-rank regularisation without training data.

Essentially, most of the existing CS-dMRI methods intend to use 2D/1D transforms to solve 3D or even higher-dimension problems. They either treat the 3D/4D data as a series of 2D images and then employ 2D/1D sparsifying transforms to explore spatial/temporal sparsity [4], or, unfold the higher-order dataset into a 2D matrix to explore the spatiotemporal redundancy [29]–[31], [35]. Intuitively, using matrix/vector transforms in dMRI data, being a higher-order tensor in nature, may not simultaneously explore the inherent data redundancy. To investigate the possibilities of preserving the higher-dimensional data format, this work proposes a novel concept of tensor sparsity for dMRI. Inspired by a recent application of the *second*-order Singular Value Decomposition (SVD) [9], [10] in exploiting in-plane sparsity, the Tucker model based Higher-order Singular Value Decomposition (HOSVD) [36], [37] was employed as a practical example for the current investigation. Tensor sparsity or tensor rank, is a powerful multidimensional signal processing tool that has been successfully applied in various areas. For instance in the area of pattern recognition/computer vision, HOSVD has been used to extract the features of the training dataset to recognise/classify future images (such as face verification) [38], [39]. Recently, a low n-rank tensor approach has also been successfully applied to dMRI to achieve high quality image reconstruction for parallel and dMRI [33]. Instead of regularising the global low-rank structure, improved reconstruction accuracy and resolution were achieved by exploiting the local low-rank structure for multidimensional MR signals, where the unknown values of the image matrices were locally estimated by considering the correlation among neighbour pixels or voxels [32],[34]. Comprehensive reviews of the applications of tensor decomposition, are provided in [40], [41]. The HOSVD in the current study takes advantage of the fact that the signals in dMRI scenario are higher-order tensors. The presented approach sparsifies the dMRI signals in their original tensor format instead of the matrix format. Three experiments were designed to present the comparisons of the performances between this tensor sparsity basis and matrix transforms. In the first and the second experiments, the *third*-order SVD (3D-SVD) was used to sparsify the cine cardiac data (two spatial dimensions plus one temporal dimension). These experiments aim to compare the performance of the proposed method for pseudo-periodical data with two existing methods in dMRI. In the third experiment, the *fourth*-order SVD (4D-SVD) was applied as sparsity basis for the dynamic volume cardiac data (three spatial dimensions plus one temporal dimension), where the feasibility of the proposed sparsity basis in 4D application is demonstrated.

The remainder of this article is organised as follows. Section 2 explains the theoretical background of the proposed method. Section 3 describes the materials and methods used for validations. Section 4 presents the comparisons of the reconstruction results between the proposed method and the existing methods. Section 5 discusses additional properties of the proposed sparsity basis. Section 6 concludes the contribution of this work.

## Theory

In this section the general formulation of dMRI reconstruction in CS framework is first introduced. Then, the construction of a key component, the spasifying transform using tensor decomposition, is described.

### 2.1. Formulation of Compressed Sensing in Dynamic MRI (CS-dMRI)

To assist the discussion, the notations of scalars, vectors, matrices (*second*-order tensors) and tensors are denoted by lowercase letters (*a*, *b*, …), capital letters (*A*, *B*, …) and calligraphic letters (** A**, ** B**, …), respectively. Letter *i* and *j* are used to index row and column vectors, respectively. (*A*)*_j_* = *A_j_* = *a_j_*, for example, denotes the *jth* column vector of matrix *A*. Hence, *A* =  (*A_1_*, *A_2_*, …,*A_J_*), where *J* is reserved for the index upper bounds, as is *I*. (*A*)*_ij_*, also symbolised as *a_ij_*, denotes the element with a row index *i* and a column index *j*.

Suppose an *Nth*-order tensor 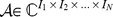 is used to represent the spatial-temporal behaviour of the imaged object. Without losing generality, the first *M* = *N*-1 dimensions of the tensor are used to describe the spatial information (for example *M* = 2 for 2D slice or *M* = 3 for 3D volume), which is collected at 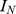 time instances. The CS-dMRI problem can be solved using the following optimisation procedure:
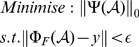
(1)where *y* is the *k* space measurements collected from the MRI scanner; *ε* represents the data-fidelity tolerance between the optimisation result and the measurements; Ψ is a transform that sparsifies the tensor ** A** (the imaging object), and Φ_F_ is a combination of operations, that is, the 2D Fourier transform for the in-plane data followed by a random under-sampling.

Equation (1) minimises the *l*_0_ *-*norm to enforce the sparsity of the object ** A**, and uses the *l*_2_ *-*norm as a constraint to guarantee the data-fidelity in the sampling domain. The optimisation problem in equation (1) is NP-hard (Non-deterministic Polynomial-time hard). The common solution for this problem is to relax the *l*_0_ norm to *l*_1_ norm, its nearest convex constraint [42]. Thus, the problem in equation (1) can be restated as:
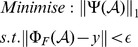
(2)


However, as has been extensively studied [43]–[48], replacing the *l_1_* norm with an *l_p_* quasi-norm (0<*p*<1) problem can reduce the amount of measurements needed for reconstruction or, can improve the reconstruction quality given the same amount of measurements. Therefore, in this work, we adopt the *l_p_* norm as a constraint to enforce the sparsity of the images. Thus, the NP-hard problem in equation (1) can be replaced by solving a problem as follows:
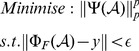
(3)where 0<*p*<1. Section 3 will describe in detail the algorithm adopted to solve the non-convex problem in the form of equation (3).

### 2.2. Construction of the Sparsity Basis Ψ using Higher-order Singular Value Decomposition

In this section, the general framework of HOSVD [37] and the applications of HOSVD as sparsifying transform in CS-dMRI will be introduced. Several higher-order tensor operations will be introduced first to pave the way for the discussion of HOSVD. The HOSVD sparsity basis was obtained from the inverse Fourier transform of the zero-filled under-sampled *k* space (denoted as 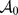), therefore no training data is required in this method.

Definition 1: Matrix unfolding-unfolding a tensor into matrices [37].

For the *Nth*-order tensor 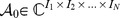, the unfolded matrix ***A*** ***_0_***_*(n)*_ contains the element 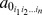 at the position with a row number 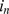 and a column number equal to
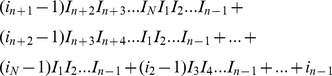



**Figure 1:**
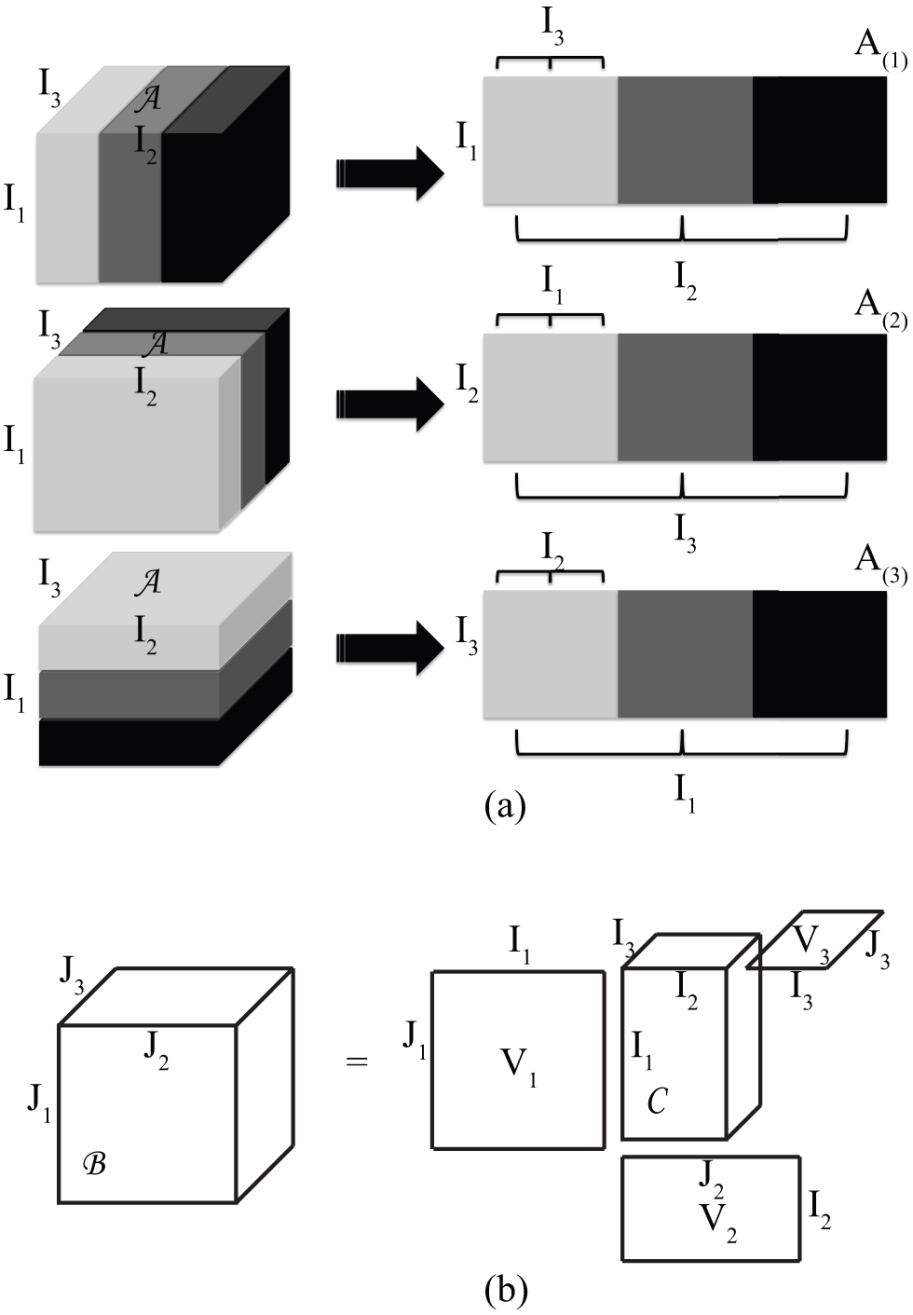
Visualisation of matrix unfolding of a tensor. (a) Visualises the matrix unfolding of a *third*-order tensor and, (b) visualises a *third*-order tensor multiplied by matrix.

Figure 1(a) exemplifies the process of unfolding a 3D tensor. There are three matrix representations (horizontal, lateral, and frontal) of the 3D tensor 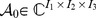 in which all the slices are stacked one after another. The lateral matrix representation 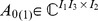 is defined as 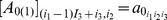; the frontal matrix representation 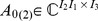
is defined as 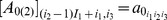; and the horizontal matrix representation 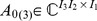 is defined as 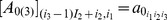.

Definition 2: Multiplication of a higher-order tensor by matrices [37].

The *n-mode* product of the tensor 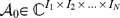 by a matrix 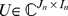, denoted as 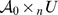 is an (*I_1_*×*I_2_*×…×*I_n-1_*×*J_n_*×*I_n+1_*×…×*I_N_*) tensor, of which the entries are given by
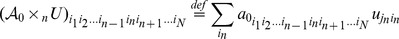



Figure 1(b) visualises the multiplication of a 3D tensor by matrix, where 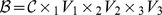 (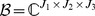,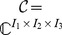). In figure 1(b) we can see that there are three multiplications of a 3D tensor by matrix. The *1-mode* product of the tensor 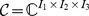 by a matrix 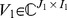 is defined as 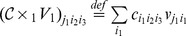, which is a 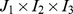 sized tensor; the *2-mode* product of 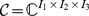 by a matrix 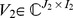 is defined as 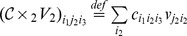, which is a 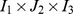 sized tensor; similarly, the *3-mode* product of 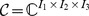 by a matrix 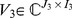 is defined as 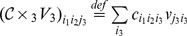, which is an 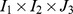 sized tensor.

With these two operations defined, any *Nth*-order tensor 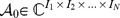 can now be decomposed, in the HOSVD framework, by
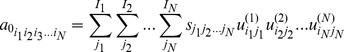
(4)where 
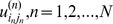
, are the entries of the unitary matrices 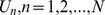, and 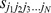 are the entries of 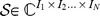 which is a complex tensor of size 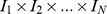. To facilitate the understanding of the properties of HOSVD, we first retrospect to the matrix SVD, which was used as a sparsity basis for static MRI [9], [10]. For any complex matrix 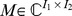, we can decompose it into product as
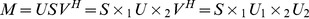
(5)where 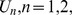 are 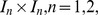 sized unitary matrices, and *S* is an 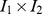 sized matrix with the properties of [37]:

- pseudo-diagonality: 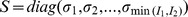

- ordering: 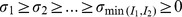

where 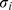 are called the singular values of *M.*

Likewise, in higher-order situation, we can decompose any complex *Nth-*order tensor 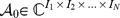 as
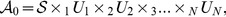
(6)where the unitary matrices 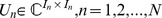 are called the *n-mode* singular matrices. Tensor 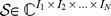 has the following properties:

- all-orthogonality: two sub-tensors 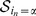 and 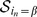 are orthogonal for all possible *n*, *α* and *β* subject to *α ≠ β*, which means 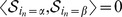 when *α ≠ β*,
- ordering: 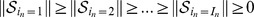 for all possible *n*,

where the Frobenius-norms 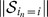, symbolised by 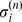, are called the *n-mode* singular values of ** A** **_0_**.

As demonstrated in [37], given a *Nth*-order tensor ** A** **_0_,** the *n-mode* singular matrix 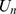 in equation (6) is actually the left singular matrix of the correlated *n-mode* matrix unfolding of ** A** **_0_** (as per Definition 1 and 2). Therefore the computation of the HOSVD in equation (6) eventually leads to *N* different matrix SVD operations on the unfolded tensor. Therefore, the tensor ** S** can be computed as
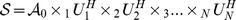
(7)


For example, *U_1_* can be obtained by performing the matrix SVD on the *1-mode* unfolding matrix 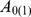 as:
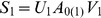
(8)


Generally, 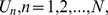 can be obtained by performing the matrix SVD on the *n-mode* unfolding matrix 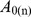 as:
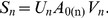
(9)


With the unitary matrices obtained, we can then construct the tensor sparsifying transform as:
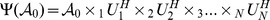
(10)where the sparsity basis 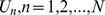 is obtained from the inverse Fourier transform of the zero-filled under-sampled *k* space 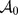.

The inverse sparsifying transform is then obtained as:
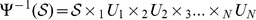
(11)


**Figure 2:**
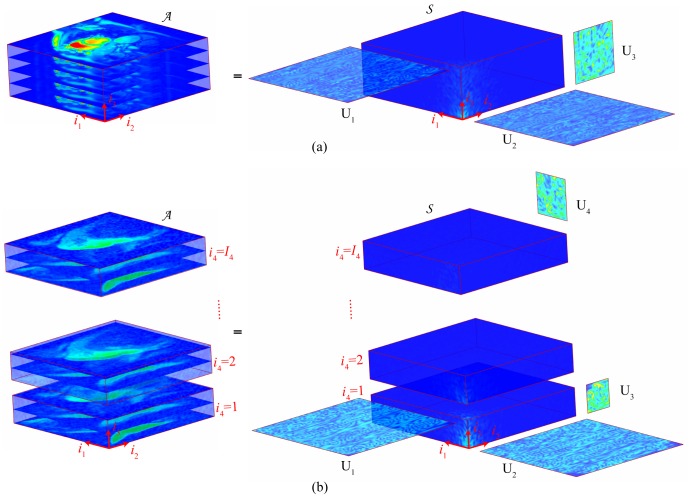
Visualisation of HOSVD. (a) Shows HOSVD on a *third*-order tensor and, (b) shows HOSVD on a *fourth*-order tensor. The left of (b) shows a four dimensional cardiac dataset denoted as **A**. The four dimensions are labelled as *i_1_*, *i_2_*, *i_3_* and *i_4_*. The right of (b) presents tensor **S** and the unitary matrices U_1_ U_2_ U_3_ and U_4_, that were obtained by performing HOSVD operation on **A**. **S** is also a *fourth-*order tensor, the dimensions of which are marked as *i_1_*, *i_2_*, *i_3_* and *i_4_*.



Figure 2(a) visualises the decomposition of a *third*-order tensor 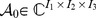 as

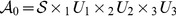
(12)The unitary matrices 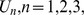 in equation (12) can be obtained from equation (9). The properties of all-orthogonality and ordering [37] guarantee that most of the energy of tensor ** S** accumulates around one vertex, and little energy distributes to the broad area away from this region. Therefore, tensor ** S** has a sparse representation (refer to figure 2(a) for illustration). Likewise, figure 2(b) presents an example of the HOSVD in the *fourth-*order tensor case. It should be noted that the tensor ** S** is shown in logarithmic scale to assist the presentation, because ** S** is too sparse to be easily visible. It is clearly shown that in both 3D and 4D cases the coefficients with large values are highly concentrated in one voxel (light blue colour), while the vast majority of the elements in the ** S** tensor are close to zero (deep blue colour).

## Materials and Methods

To test the possibility of employing HOSVD as higher-order sparsifying transform in CS-dMRI applications, three experiments are designed: two cine cardiac MRI schemes and one dynamic volume cardiac MRI series.

### 3.1. Datasets

#### 3.1.1. 3D-SVD: Application in cine cardiac MRI

**Figure 3:**
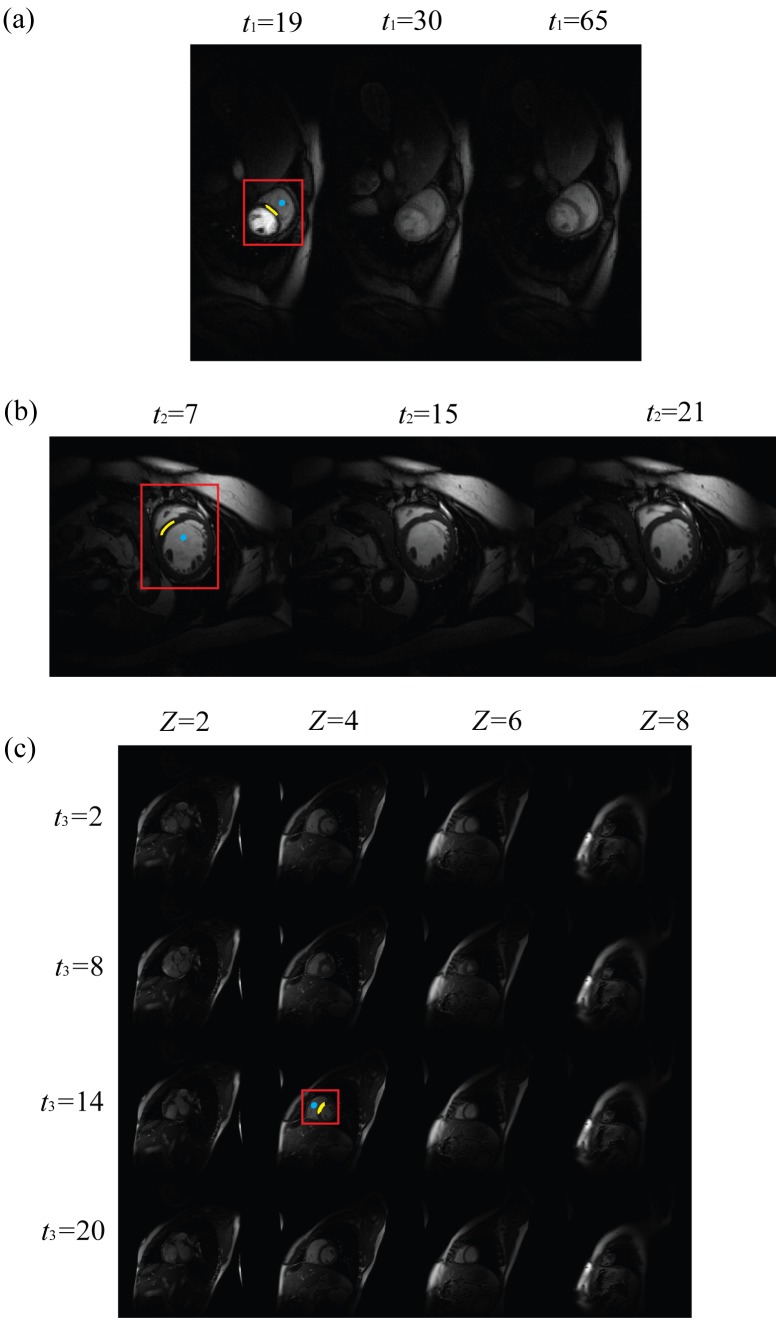
Several frames of the datasets obtained at different time instants (as indicated). From top to bottom: (a) Dataset A, (b) Dataset B and, (c) Dataset C. In (c), images obtained at four time instants (as indicated by *t_3_*) are presented row by row, and images obtained at four *z* positions (as indicated by *z,* along axial direction (base-apex)) are presented column by column. The regions of interest of each dataset are marked within the red rectangles and, the regions of myocardial and blood pool used for averaged signal intensity comparison are marked with yellow and light blue colours, respectively.

Two sets of cine cardiac MRI data were used to validate the proposed method. The first dataset (Dataset A) was acquired at the University of Utah, which was used in the method k-t SLR [30]. 70 frames of k-space were acquired on a 3T Siemens scanner with the spatial resolution of 90×190 (phase encoding×frequency encoding). The cardiac data was obtained with a saturation recovery sequence (TR/TE = 2.5/1 ms, saturation recovery time = 100 ms). The second dataset (Dataset B) was acquired at Yonsei University Medical Center, which was used in the method k-t FOCUSS [12], [13]. 25 frames of full k-space data was acquired using a 1.5T Philips system with an in-plane spatial resolution of 256×256. The cine cardiac data was obtained using steady-state free precession (SSFP) sequence with a flip angle of 50 degree and TR = 3.45 msec. The FOV was 345 mm×270 mm. The slice thickness was 10 mm. A few frames from both Dataset A and Dataset B are shown in figure 3(a, b).

#### 3.1.2. 4D-SVD: Application in volume dynamic cardiac MRI

The third experiment investigated the possibility of employing the proposed HOSVD sparsifying transform for 4D dynamic cardiac MRI. The images of the this dataset (Dataset C) were of one subject, arbitrarily chosen from a total of 33 available subjects [49]. The measurements were acquired from a GE Genesis Signa MR scanner using the FIESTA protocol. The dimension of the subject data is 256×256×10×20 (phase encoding×frequency encoding×z position×time). It is noted that Dataset C is in DICOM (Digital Imaging and Communications in Medicine) format. Using the real-valued images, instead of the complex-valued k-space data, has made the reconstruction of the 4D experiment easier. A few frames from Dataset C are shown in figure 3(c).

### 3.2. Reconstruction

#### 3.2.1. Optimisation algorithm

The *l_p_* quasi-norm in equation (3) poses a non-convex optimisation problem. Theoretical work [44], [46] has demonstrated that this non-convex problem is solvable and, the local minima can be avoided in practice [43], [50]. The applications in the medical imaging context [30], [45], [47], [48], [51]–[55], have already demonstrated the practicability and advances of non-convex optimisation. In this work, we adopt the algorithms used in [30], [47] to solve the optimisation problem stated in equation (3). In [47], Chartrand used both wavelet transform and discrete gradient to enforce the sparsity of the MR images. In [30], Lingala et al. used the combination of rank property and signal sparsity for reconstruction. In this work we use only the HOSVD for sparsity enforcement. Therefore we herein briefly state the modified optimisation process as follows.

We begin with the definition of a variable splitting operator:
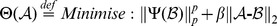
(13)where 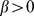 is a constant. It is noted that 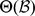 is forced to approach 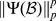 when 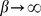.

We rewrite the problem in equation (3) as into its Lagrange’s form as:
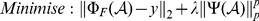
(14)where 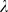 is a constant to balance the weighting between the data fidelity and the signal sparsity. Then the splitting operator was applied on equation (14), arriving at:



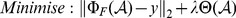
(15)which can be expanded as:
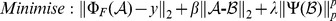
(16)


We can then solve the problem above by iteratively solving the variables ** A** and ** B** in turn. In this way the problem in equation (16) is decomposed into two simple sub-problems. The two sub-problems are decoupled, making it computational efficient. By setting 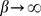, the solution of equation (16) approaches that of equation (14).

To solve the sub-problem with respect to variable ** A**, we can fix variable ** B** and adopt the conjugate gradient algorithm as used in [30]:
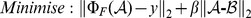
(17)


To solve the sub-problem with respect to variable ** B**, we fix variable ** A** and apply *p-*shrinkage operator to each pixel of 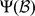. As explained in [47] the *p-*shrinkage operator is executed as:
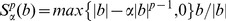
(18)


**Figure 4:**
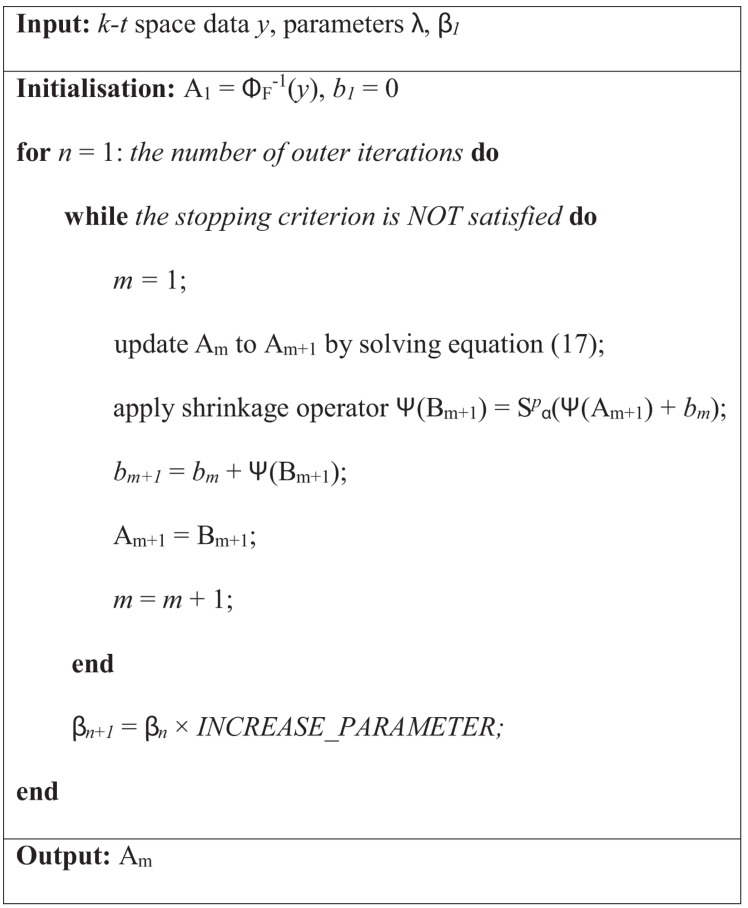
Outline of the reconstruction algorithm.

To choose an appropriate value for the parameter *β*, we initialised it with a relatively small value and then geometrically increased it as proposed in [56]. To enforce the data-fidelity, the residual of each sub-problem was added back to the data at each iteration as proposed in [57]. For a summary of the optimisation, please refer to figure 4.

### 3.3. Comparison Validations

The proposed method was compared with one of the recent low-rank image reconstruction methods, *k-t* SLR, and a classic CS method, *k-t* SPARSE. For fair comparison, we ensure that firstly all the methods used the same sampling pattern of *k-t* space; secondly, the parameters for all the methods were adjusted appropriately so that both the signal to error ratio (SER) and the averaged signal intensity for all methods were optimised; and thirdly the optimisations for all the methods share the same stopping criterion, that is the optimisations ceased when the gradient magnitude of the object function reached 1×10^−4^ or the number of iterations reached 300. All the evaluations were implemented using Matlab 2011a (MathWorks, Natick, MA) on a Mac OS X Lion operation system, with a dual-core 2.4 GHz Intel processor and 4 GB of memory. The SER was calculated as:
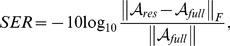
(19)where ** A** *_res_* is the result of the reconstruction, ** A** *_full_* is the fully sampled dynamic images, and 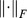 denotes the Frobenius norm. A greater SER value correlates to a better image quality.

The method *k-t* SLR employs two regularisations: the low-rank structure and the sparsity of the signal. To exploit the low-rank structure, *k-t* SLR reshaped the 3D dataset into a large 2D matrix **Γ**. More specifically, the 2D images in a dynamic sequence were firstly vectorised and then concatenated to form the matrix **Γ**. To exploit the sparsity of the signal, the total variation (TV) was used as an extra regularisation. Moreover, instead of using convex penalties to regularise the low rank property and the sparsity, *k-t* SLR adopted some of the recent algorithms on the non-convex regularisation [43], [47], [48] for the optimisation, further improving the reconstruction result. In [30], the combination of the constraints provided better image quality than the variants of the *k-t* SLR, which rely on either matrix SVD or TV constraint alone. Therefore in this work, we only compare the proposed method with *k-t* SLR, where both SVD and the TV regularisations were used in the optimisation. The method *k-t* SPARSE is a classic CS-dMRI method. It uses the wavelet transform (Daubechies 4 was used as the mother wavelet in this work) for in-plane sparsity and the Fourier transform for temporal sparsity, assuming that the change of the heartbeat is periodical. All the methods compared in this work are flexible to account for arbitrary non-Cartesian *k-*space sampling schemes; we adopt the radial trajectory with uniform angular spacing as used in [30]. The trajectory was randomly rotated with a small angle for each frame to implement random sampling.

## Results

### 4.1. 3D Application

**Figure 5:**
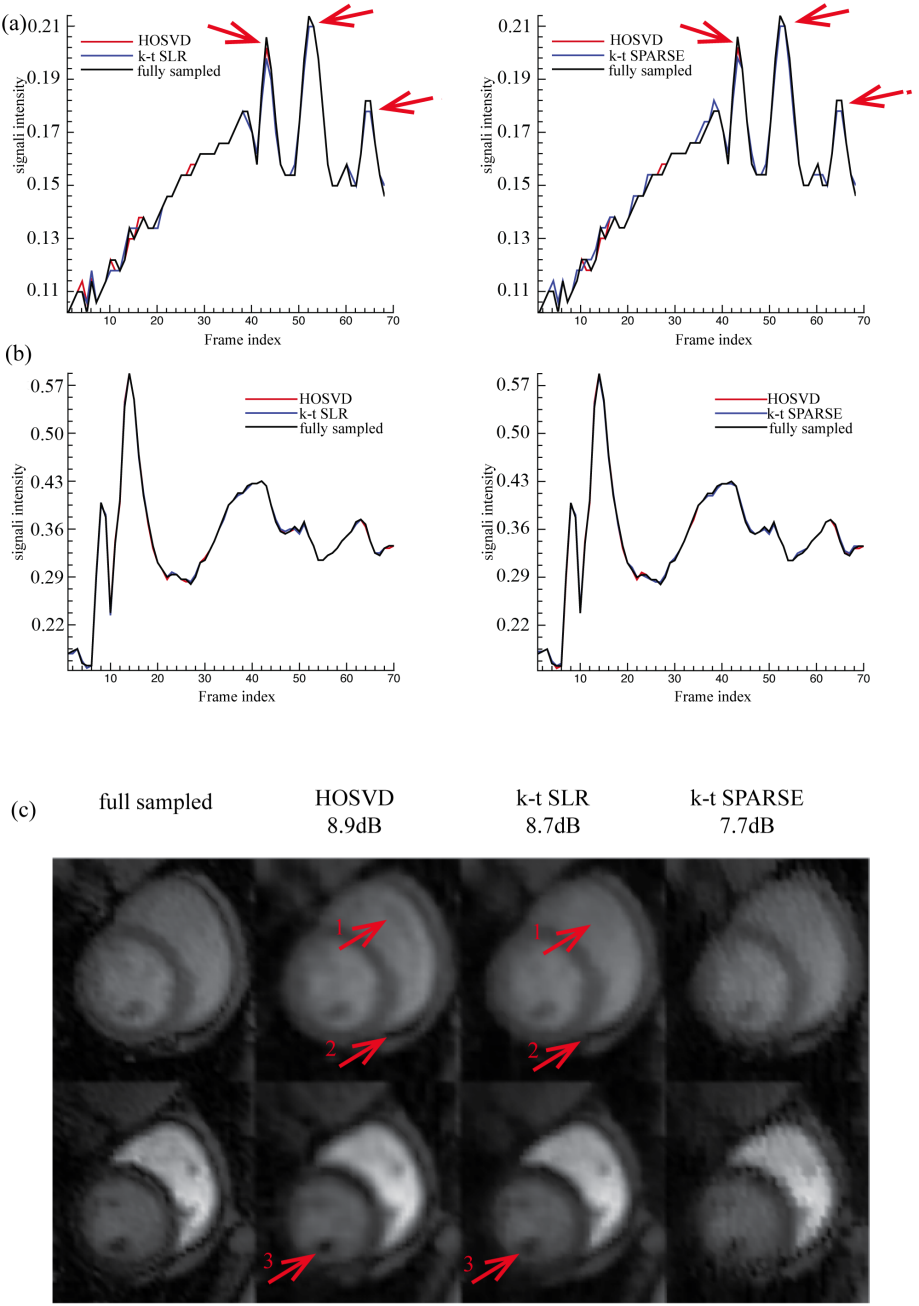
Reconstructions of Dataset A at reduction factor 6. (a) and (b) show the averaged normalised signal intensity at the myocardial and blood pool regions, respectively, and (c) shows the images (region of interest only) at the peak signal intensity of myocardial (the 54^th^ frame, top row) and blood pool (the 14^th^ frame bottom row). The left of (a) and (b) shows the averaged signal intensity of the fully sampled images (black line), *k-t* SLR reconstruction (blue line) and, the reconstruction of the proposed method (red line); the right of (a) and (b) shows the averaged signal intensity of the fully sampled images (black line), the *k-t* SPARSE reconstruction (blue line) and, the reconstruction of the proposed method (red line).

**Figure 6:**
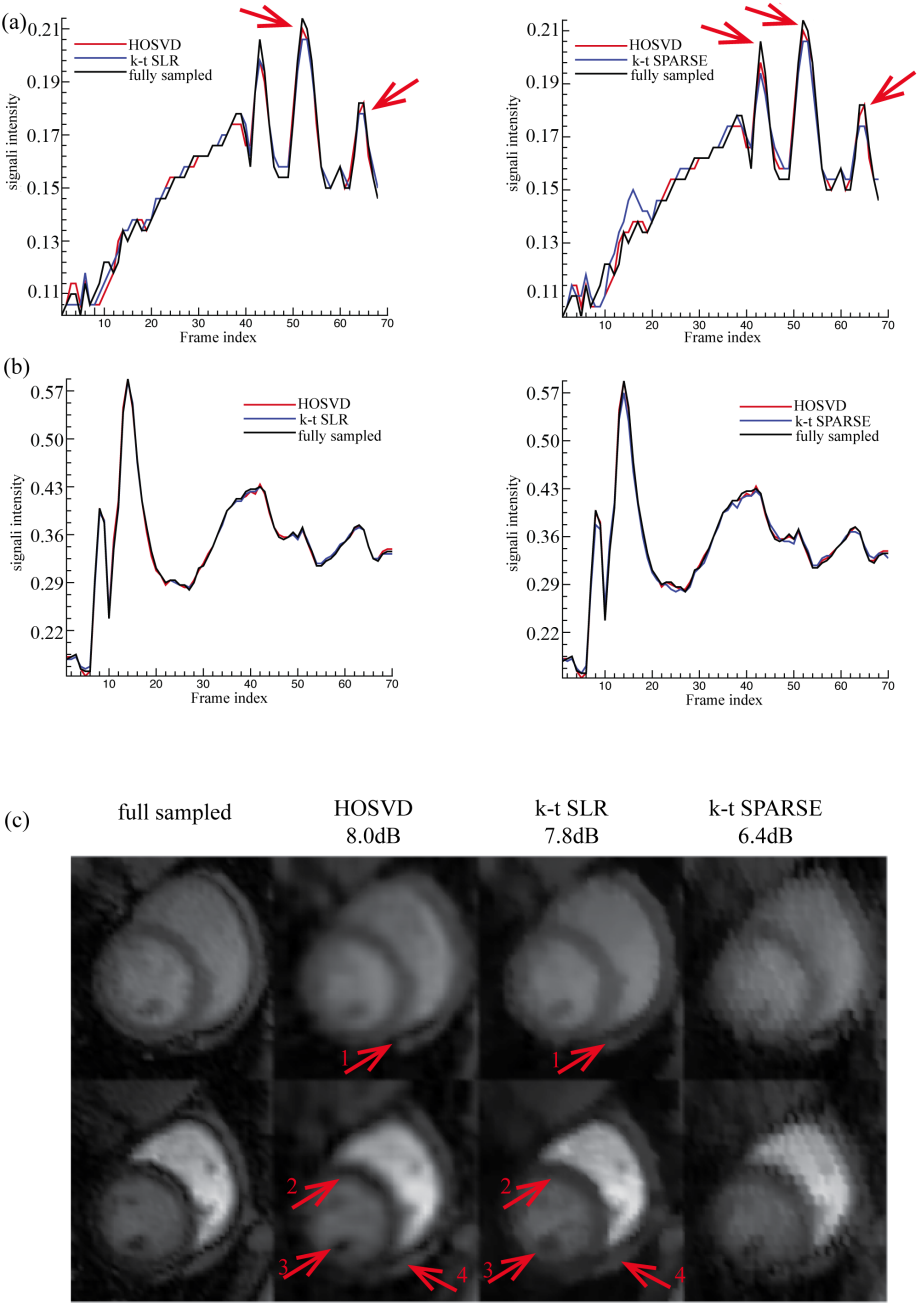
Reconstructions of Dataset A at reduction factor 11. (a) and (b) show the averaged normalised signal intensity at the myocardial and blood pool regions, respectively, and (c) shows the images (region of interest only) at the peak signal intensity of myocardial (the 54^th^ frame, top row) and blood pool (the 14^th^ frame bottom row). The left of (a) and (b) shows the averaged signal intensity of the fully sampled images (black line), *k-t* SLR reconstruction (blue line) and, the reconstruction of the proposed method (red line); the right of (a) and (b) shows the averaged signal intensity of the fully sampled images (black line), the *k-t* SPARSE reconstruction (blue line) and, the reconstruction of the proposed method (red line).

**Figure 7:**
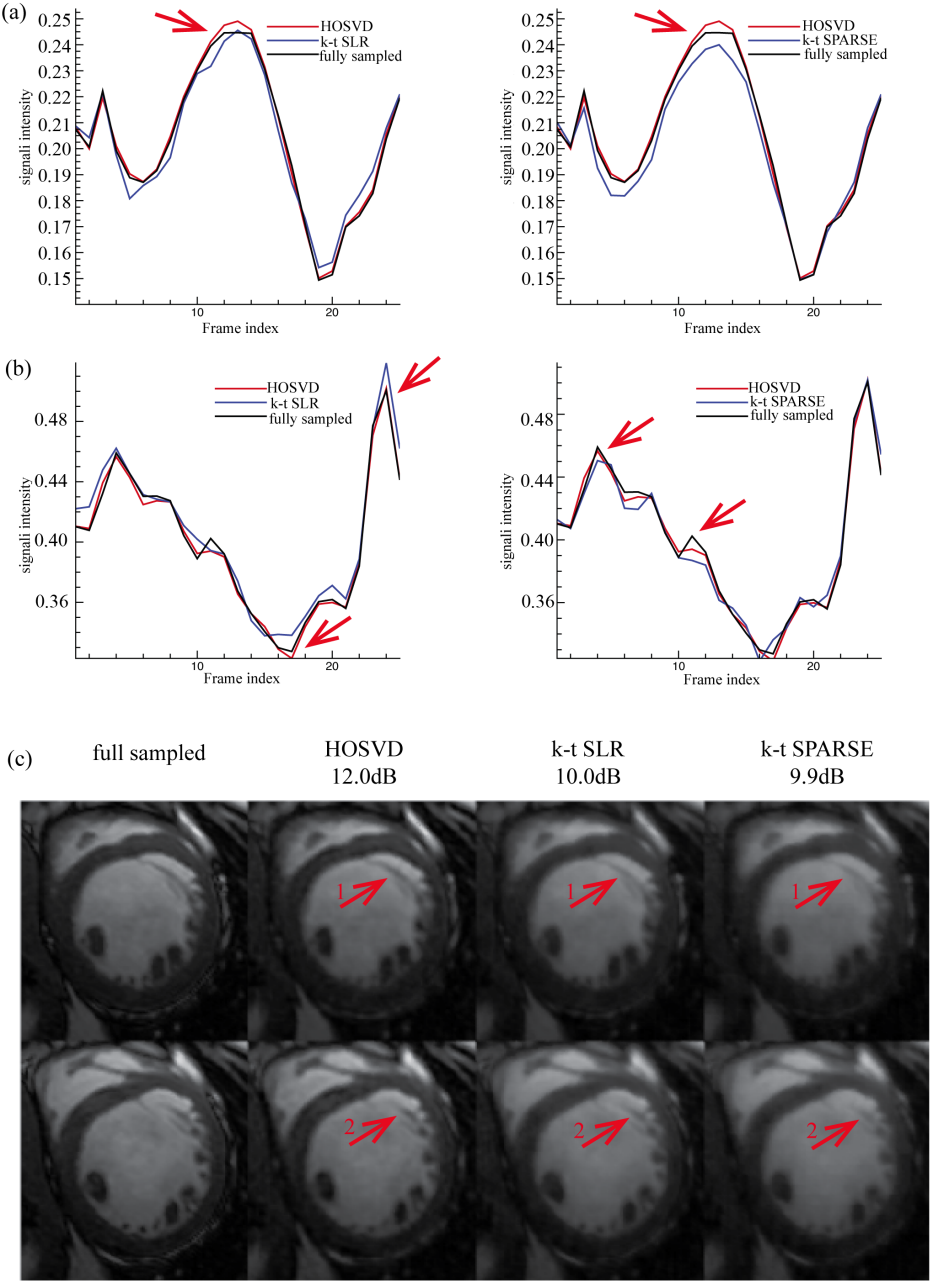
Reconstructions of Dataset B at reduction factor 6. (a) and (b) show the averaged normalised signal intensity at the myocardial and blood pool regions, respectively, and (c) shows the images (region of interest only) at the peak signal intensity of myocardial (the 13^th^ frame, top row) and blood pool (the 24^th^ frame bottom row). The left of (a) and (b) shows the averaged signal intensity of the fully sampled images (black line), *k-t* SLR reconstruction (blue line) and, the reconstruction of the proposed method (red line); the right of (a) and (b) shows averaged the signal intensity of the fully sampled images (black line), the *k-t* SPARSE reconstruction (blue line) and, the reconstruction of the proposed method (red line).

**Figure 8:**
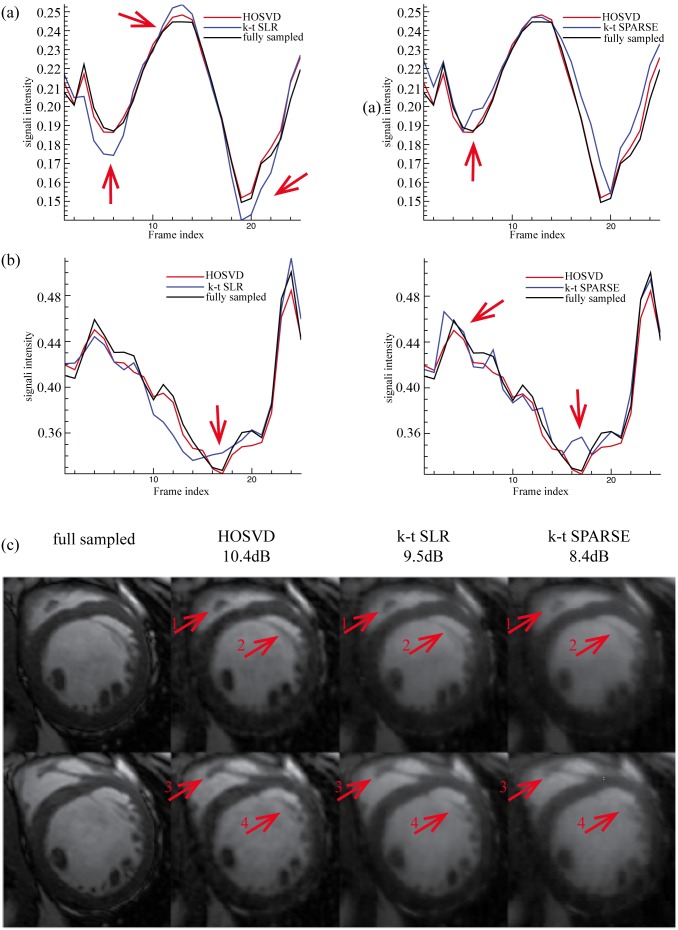
Reconstructions of Dataset B at reduction factor 11. (a) and (b) show the averaged normalised signal intensity at the myocardial and blood pool regions, respectively, and (c) shows the images (region of interest only) at the peak signal intensity of myocardial (the 13^th^ frame, top row) and blood pool (the 24^th^ frame bottom row). The left of (a) and (b) shows the averaged signal intensity of the fully sampled images (black line), *k-t* SLR reconstruction (blue line) and, the reconstruction of the proposed method (red line); the right of (a) and (b) shows the averaged signal intensity of the fully sampled images (black line), the reconstruction of *k-t* SPARSE (blue line) and, the reconstruction of the proposed method (red line).

**Figure 9:**
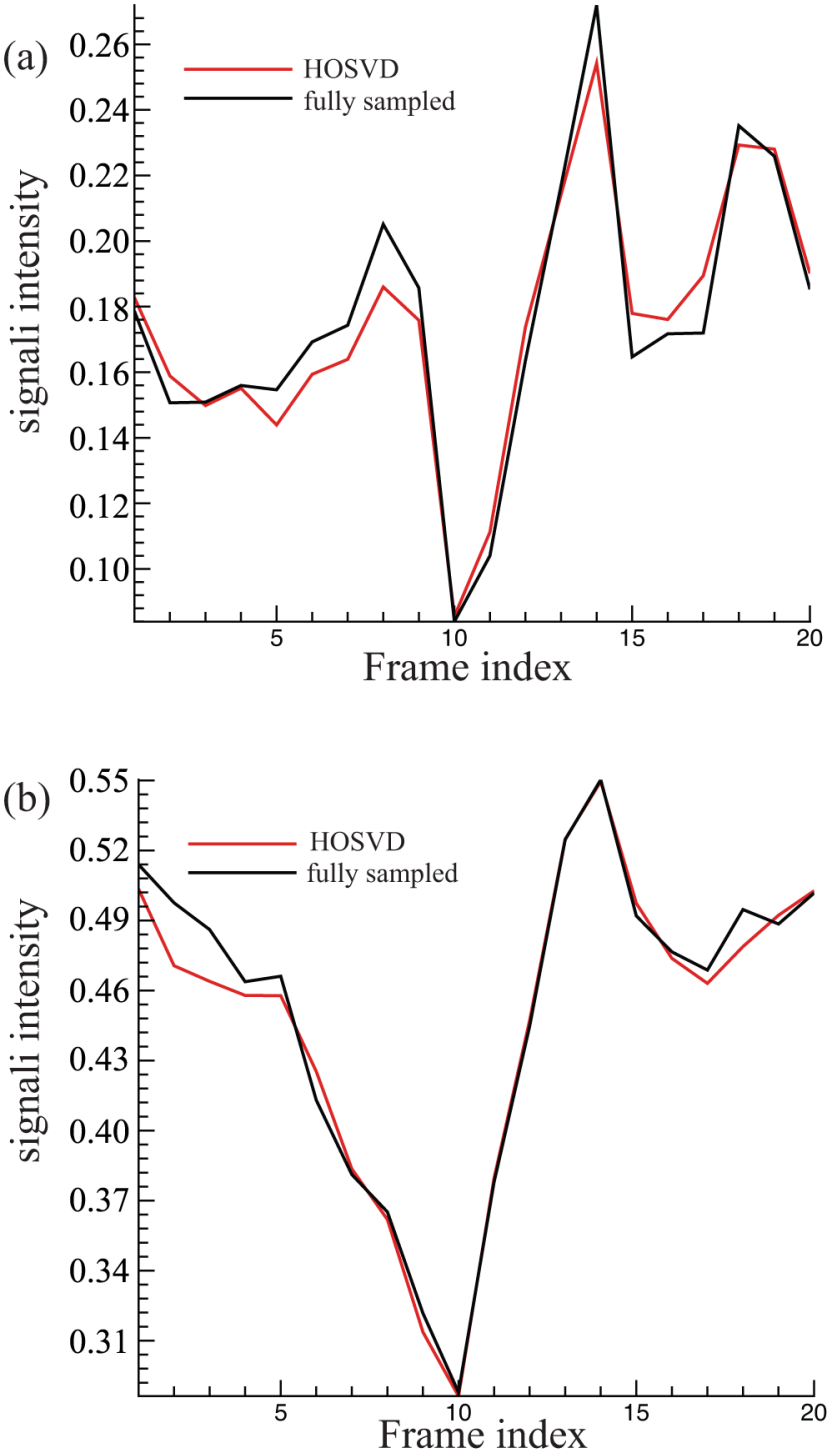
The averaged normalised signal intensity achieved by the proposed method at reduction factor 11. (a) The myocardial signal intensity of the fully sampled images and the reconstructed images provided by the proposed method; (b) the blood pool signal intensity of the fully sampled images and the reconstructed images provided by the proposed method.

Figure 5 and 6 show the reconstruction of Dataset A at reduction factors 6 and 11 respectively. When the reduction factor was 6 (reduction factor *n* means only *1/n* of the full *k-*space measurements were obtained), the SER values achieved by the proposed method, the *k-t* SLR and the *k-t* SPARSE, were 8.9 dB, 8.7 dB and, 7.7 dB, respectively. Figure 5 shows the reconstruction of Dataset A when the reduction factor was 6. As shown in figure 5(b), all the methods provided comparable averaged signal intensity for the blood pool area (normalised to the maximum grey level of the region of interest, figures 6, 7, 8, and 9 are normalised in the same fashion). However, when comparing at the myocardial signal intensity, both the proposed method and *k-t* SLR obviously outperformed *k-t* SPARSE, especially at the frames where the averaged signal intensity changed rapidly (as marked with red arrows in figure 5(a)). The region of interest (as marked in figure 3(a)) of the 54^th^ and the 14^th^ frames, where the myocardial and the blood pool signal intensities reached their peak values, are presented on the top and the bottom rows of figure 5(c), respectively. In figure 5(c), it appears that Dataset A contains visible white noise, and some of the residual noise was maintained in the result of the method *k-t* SPARSE. The images recovered by the proposed method and the *k-t* SLR successfully supressed the white noise. Both the proposed method and the *k-t* SLR provided comparable overall image quality at the low reduction factor. When the reduction factor was 11, the SER values achieved by the proposed method, the *k-t* SLR and the *k-t* SPARSE, were 8.0 dB, 7.8 dB and, 6.4 dB, respectively. As shown in figure 6(b), all the methods recovered comparable averaged signal intensity of the blood pool area. However, when comparing the myocardial area, the proposed method and the *k-t* SLR outperformed the *k-t* SPARSE more obviously, especially at the frames where the signal changes quickly (as indicated by the red arrows in figure 6(a)). The region of interest of the 54^th^ and the 14^th^ images are presented on the top and the bottom row of figure 6(c), respectively. The *k-t* SPARSE was severely affected by the white noise at this high reduction factor, while both the *k-t* SLR and the proposed method were still robust to noise. In *k-t* SLR, TV regularisation provided slightly better reconstruction for large contours or boundaries of the images. However, it also generated a cartoon-like/over-smooth effect on local fine details (also observed in [30]). This effect is more obvious at reduction factor 11 (see figure 6(c)). Compared with TV regularised *k-t* SLR, the proposed method provided slightly better reconstruction of local fine details (see the red arrows in figure 6(c)). The evaluation of all the methods based on Dataset A indicates that the proposed tensor sparsity basis outperformed the conventional matrix sparsity basis. Moreover, even when comparing with *k-t* SLR that combines the low-rank matrix recovery and the sparsity constraint, the proposed method was still able to provide comparable overall reconstruction accuracy.

Figure 7 and 8 show the reconstruction of Dataset B provided by all the methods at reduction factors of 6 and 11, respectively. When the reduction factor was 6, the proposed method, the *k-t* SLR and the *k-t* SPARSE achieved the SER values of 12dB, 10dB and, 9.9dB, respectively. The averaged signal intensity comparison, as shown in figure 7(a, b), demonstrates that the proposed method was more capable of capturing the dynamic features of the signal (see the red arrows in figure 7(a, b)) than *k-t* SPARSE. The 13^th^ and the 24^th^ frames (region of interest only, as marked in figure 3(b)), where the peak averaged signal intensity of myocardial and blood pool areas were reached, are presented on the top and the bottom rows of figure 7(c), respectively. As shown in figure 7 (c), Dataset B contains more local details than Dataset A and, it has little visible white noise. All the methods succeeded in recovering the coarse features of Dataset B; meanwhile, the proposed method and the *k-t* SLR captured more fine details (see the red arrows in figure 7(c)). When the reduction factor was 11, the SER values achieved by the proposed method, the *k-t* SLR and the *k-t* SPARSE, were 10.4 dB, 9.5 dB and, 8.4 dB, respectively. The averaged signal intensity of the myocardial and the blood pool was compared in figure 8(a, b). The proposed method achieved comparable reconstruction with the *k-t* SLR and, better overall reconstructions as compared to *k-t* SPARSE. And the visual evaluation in figure 8(c) shows consistent results with those of the averaged signal intensity comparison, as indicated by the red arrows. The quantitative and visual evaluations of Dataset B were also consistent with those of Dataset A.

### 4.2. 4D Application

**Figure 10:**
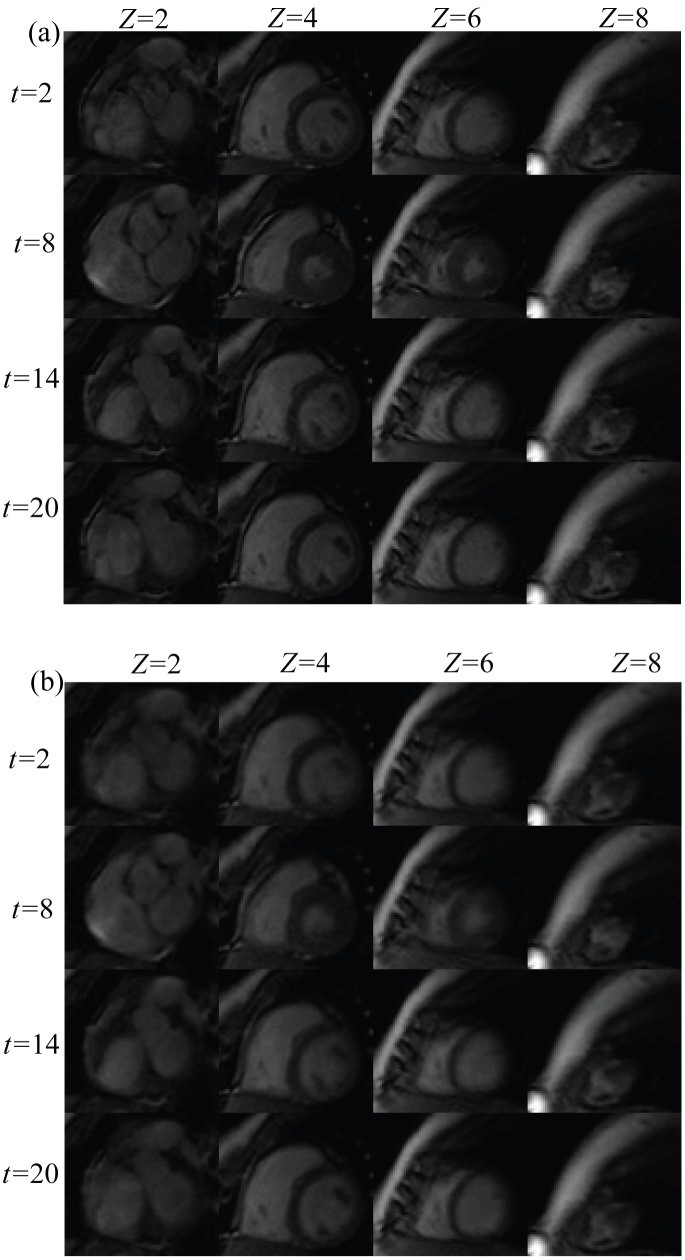
The reconstruction of Dataset C (region of interest only) achieved by the proposed method at reduction factor 11. (a) Presents several fully sampled images, and (b) presents the corresponding reconstructed images. In (a), images obtained at four time instants (indicated by *t*) are present row by row; and images obtained at four *z* positions (indicated by *z* along axial direction (base-apex)) are presents column by column. Likewise, (b) presents the reconstructed images at the corresponding time instants (indicated by *t*) and *z* positions (indicated by *z*).

As shown in figure 2, the HOSVD method can be applied straightforwardly to higher order datasets. In this work, we present the application of HOSVD in the dynamic volume cardiac imaging, where the dataset is a 4D tensor. At reduction factor 11, the SER of the reconstructed 4D images achieved by the proposed method was 12.1 dB. The averaged signal intensity at the myocardial and the blood pool areas is presented in figure 9. As illustrated in figure 9, at the high reduction factor of 11 the proposed method was still able to recover the dynamic features of the signal without noticeable error. Several fully sampled and the reconstructed images (region of interest only, as marked in figure 3(c)) are shown in figure 10(a) and (b), respectively. As shown in figure 10(b), the proposed method successfully recovered the coarse features of the object and, most of the fine details were also recovered, which demonstrated the feasibility of the proposed sparsifying transform for the 4D application.

## Discussions

### 5.1. The Tucker Model Based HOSVD

This work takes the Tucker model based HOSVD as an example to demonstrate the potential of tensor decomposition in the exploration of higher-order signal sparsity. The Tucker model based HOSVD decomposes a dense tensor into a sparse tensor multiplied by matrices along individual modes (as shown in figure (1–2)). The *k-t* SLR actually used solely the *mode-2* unfold of the tensor structure to explore the low rank properties. However, this work does not explore the low-rank structure of the reshaped tensor. Instead, it explores the sparsity in a tensor structure. In addition to HOSVD, there are a broad range of tensor decomposition techniques for future investigation, such as the CANDECOMP/PARAFAC decomposition [58], [59] and its variants, which can be used to explore the tensor rank minimisation.

### 5.2. Computational Cost

In this work, when the same stopping criteria was set, the computation time for the proposed method in the 3D application was, on average, 28 and 29 minutes for Dataset A and B respectively. The *k-t* SLR method used 21 minutes for Dataset A and 29 minutes for Dataset B. As for the *k-t* SPARSE, it took 29 minutes on average for Dataset A and 40 minutes for Dataset B. In dealing with *third*-order tensor, the proposed method performs SVD three times (once per equation (8–10)), while *k-t* SLR needs only one SVD computation. The proposed method involves only one regularisation, while the *k-t* SLR involves two regularisations. Therefore, though the computing time of the HOSVD basis function is three times that of the SVD basis function, the overall optimisation time of the proposed method was only approximately 30% more than that of the *k-t* SLR. The sparsifying transform in the *k-t* SPARSE involves multiple times of wavelet transforms for each frame and one Fourier transform for the temporal dimension therefore, it was slightly slower than the proposed method.

### 5.3. Parameter Setting

The balance between the data fidelity in *k*-space and the sparsity of the images has become a common concern in many the CS approaches. This issue becomes more complicated when more than one regularisation terms are involved in the optimisation, such as in the method *k-t* SLR. Although the setting of the regularisation parameters has been discussed within the CS-MRI framework [6060,61], it is believed that further investigation is still required for specific applications. As far as we have observed, when using the same sparsifying transform, the image artefacts increase as the amount of *k*-space acquisitions decreases. Therefore, the weighting of the sparsity constraint needs to be slightly increased. With the same reduction factor, different sparsifying transforms provide significantly different values of the *l_p_* quasi-norm, while the values of the *l_2_* norm (the data-fidelity in *k-*space) stay relatively stable. Therefore it would be inappropriate if the values of *λ* are identical for different sparsifying transforms. Instead they should be optimised case by case.

## Conclusion

This work proposes a novel concept of tensor sparsity for Compressed Sensing in dynamic MRI, and presents the Tucker model based Higher-order Singular Value Decomposition as a practical example. The tensor decomposition based method derives the sparsity basis adaptively and directly from the zero-filled under-sampled *k-t* space measurements, and does not require extra scan time to obtain training data. The proposed tensor sparsity basis provides improved image reconstruction quality when compared to the classic sparsity basis. The reconstruction quality is similar to that with a stronger constraint–low rank property of matrix.

## References

[1] Haase A (1990) Snapshot flash mri. applications to t1, t2, and chemical-shift imaging. Magnetic Resonance in Medicine 13: 77–89. 2319937 10.1002/mrm.1910130109

[2] Stehling MK, Turner R, Mansfield P (1991) Echo-planar imaging: magnetic resonance imaging in a fraction of a second. Science 254: 43–50. 1925560 10.1126/science.1925560

[3] Donoho DL (2006) Compressed sensing. IEEE Transactions on Information Theory 52: 1289–1306.

[4] Lustig M, Santos JM, Donoho DL, Pauly JM (2006) kt SPARSE: High frame rate dynamic MRI exploiting spatio-temporal sparsity. Proceedings of the 13th Annual Meeting of ISMRM, Seattle. 2420.

[5] Lustig M, Donoho DL, Santos JM, Pauly JM (2008) Compressed sensing MRI. IEEE Signal Processing Magazine 25: 72–82.

[6] Candes EJ, Romberg J (2007) Sparsity and incoherence in compressive sampling. Inverse Problems 23: 969.

[7] Candes EJ, Romberg J, Tao T (2006) Robust uncertainty principles: exact signal reconstruction from highly incomplete frequency information. IEEE Transactions on Information Theory 52: 489–509.

[8] Lustig M, Donoho D, Pauly JM (2007) Sparse MRI: The application of compressed sensing for rapid MR imaging. Magnetic Resonance in Medicine 58: 1182–1195. 17969013 10.1002/mrm.21391

[9] Hong M, Yu Y, Wang H, Liu F, Crozier S (2011) Compressed sensing MRI with singular value decomposition-based sparsity basis. Physics in Medicine and Biology 56: 6311–6325. 21896962 10.1088/0031-9155/56/19/010

[10] Yu Y, Hong M, Liu F, Wang H, Crozier S (2011) Compressed sensing MRI using Singular Value Decomposition based sparsity basis. Engineering in Medicnie and Biology Society, EMBC, 2011 Annual International Conference of the IEEE on. Boston USA. Aug. 30 2011-Sept. 3 2011: 5734–5737. 10.1109/IEMBS.2011.6091419 22255642

[11] Leonid R, Stanley O, Emad F (1992) Nonlinear total variation based noise removal algorithms. Phys D 60: 259–268.

[12] Jung H, Ye JC, Kim EY (2007) Improved k-t BLAST and k-t SENSE using FOCUSS. Physics in medicine and biology 52: 3201–3226. 17505098 10.1088/0031-9155/52/11/018

[13] Jung H, Sung K, Nayak KS, Kim EY, Ye JC (2009) k-t FOCUSS: A general compressed sensing framework for high resolution dynamic MRI. Magnetic Resonance in Medicine 61: 103–116. 19097216 10.1002/mrm.21757

[14] Gorodnitsky IF, George JS, Rao BD (1995) Neuromagnetic source imaging with FOCUSS: a recursive weighted minimum norm algorithm. Electroencephalography and clinical neurophysiology 95: 231–251. 8529554 10.1016/0013-4694(95)00107-a

[15] Gorodnitsky IF, Rao BD (1997) Sparse signal reconstruction from limited data using FOCUSS: a re-weighted minimum norm algorithm. Signal Processing, IEEE Transactions on 45: 600–616.

[16] Liang D, DiBella EVR, Chen R-R, Ying L (2011) K-T ISD: Compressed sensing with iterative support detection for dynamic MRI. Biomedical Imaging: From Nano to Macro, 2011 IEEE International Symposiumon: IEEE. 1264–1267.

[17] Akçakaya M, Basha TA, Goddu B, Goepfert LA, Kissinger KV, et al (2011) Low-dimensional-structure self-learning and thresholding: Regularization beyond compressed sensing for MRI Reconstruction. Magnetic Resonance in Medicine 66: 756–767. 21465542 10.1002/mrm.22841 PMC4212512

[18] Prieto C, Usman M, Wild JM, Kozerke S, Batchelor PG, et al (2012) Group sparse reconstruction using intensity-based clustering. Magnetic Resonance in Medicine 69: 1169–1179. 22648740 10.1002/mrm.24333

[19] Candès EJ, Recht B (2009) Exact matrix completion via convex optimization. Foundations of Computational mathematics 9: 717–772.

[20] Meka R, Jain P, Dhillon IS (2009) Guaranteed rank minimization via singular value projection. arXiv preprint arXiv: 09095457.

[21] Candes EJ, Plan Y (2010) Matrix completion with noise. Proceedings of the IEEE 98: 925–936.

[22] Keshavan RH, Montanari A, Oh S (2010) Matrix completion from noisy entries. The Journal of Machine Learning Research 99: 2057–2078.

[23] Dai W, Milenkovic O (2010) SET: an algorithm for consistent matrix completion. Acoustics Speech and Signal Processing (ICASSP), 2010 IEEE International Conference on. Dallas USA. 3646–3649.

[24] Lee K, Bresler Y (2010) Admira: Atomic decomposition for minimum rank approximation. Information Theory, IEEE Transactions on 56: 4402–4416.

[25] Recht B, Fazel M, Parrilo PA (2010) Guaranteed minimum-rank solutions of linear matrix equations via nuclear norm minimization. SIAM review 52: 471–501.

[26] Brinegar C, Wu YJL, Foley LM, Hitchens TK, Qing Y, et al (2008) Real-time cardiac MRI without triggering, gating, or breath holding; 20–25 Aug. 2008: 3381–3384. 10.1109/IEMBS.2008.4649931 PMC2793087 19163434

[27] Brinegar C, Haosen Z, Wu YJL, Foley LM, Hitchens TK, et al (2009) Real-time cardiac MRI using prior spatial-spectral information. Engineering in Medicnie and Biology Society, EMBC, 2009 Annual International Conference of the IEEE on. Minneapolis USA. 3–6 Sept. 2009: 4383–4386. 10.1109/IEMBS.2009.5333482 PMC2800039 19964109

[28] Pedersen H, Kozerke S, Ringgaard S, Nehrke K, Kim WY (2009) k-t PCA: Temporally constrained k-t BLAST reconstruction using principal component analysis. Magnetic Resonance in Medicine 62: 706–716. 19585603 10.1002/mrm.22052

[29] Haldar JP, Liang Z-P (2010) Spatiotemporal imaging with partially separable functions: a matrix recovery approach. Biomedical Imaging: From Nano to Macro, 2010 IEEE International Symposium on. Rotterdam Netherlands. 716–719.

[30] Lingala SG, Hu Y, DiBella E, Jacob M (2011) Accelerated dynamic MRI exploiting sparsity and low-rank structure: k-t SLR. IEEE Transactions on Medical Imaging 30: 1042–1054. 21292593 10.1109/TMI.2010.2100850 PMC3707502

[31] Haldar JP, Liang Z-P (2011) Low-rank approximations for dynamic imaging. Biomedical Imaging: From Nano to Macro, 2011 IEEE International Symposium on. Chicago USA. 1052–1055.

[32] Trzasko J, Manduca A, Borisch E (2011) Local versus global low-rank promotion in dynamic MRI series reconstruction. Proceeding of the 19th Annual Meeting of ISMRM. Montréal, Canada. 4371.

[33] Trzasko J, Manduca A (2013) A Unified Tensor Regression Framework for Calibrationless Dynamic, Multi-Channel MRI Reconstruction; 2013; Salt Lake City, USA.

[34] Trzasko JD (2013) Exploiting local low-rank structure in higher-dimensional MRI applications. International Society for Optics and Photonics. Proc. SPIE 8858, Wavelets and Sparsity XV, 885821 (September 26, 2013). doi:10.1117/12.2027059.

[35] Liang Z-P (2007) Spatiotemporal imaging with partially separable functions. Noninvasive Functional Source Imaging of the Brain and Heart and the International Conference on Functional Biomedical Imaging, 2007. NFSI-ICFBI 2007. Joint Meeting of the 6th International Symposium on. Hangzhou China. 181–182.

[36] Tucker LR (1966) Some mathematical notes on three-mode factor analysis. Psychometrika 31: 279–311. 5221127 10.1007/BF02289464

[37] De Lathauwer L, De Moor B, Vandewalle J (2000) A multilinear singular value decomposition. SIAM Journal on Matrix Analysis and Applications 21: 1253–1278.

[38] Wang S-J, Yang J, Sun M-F, Peng X-J, Sun M-M, et al (2012) Sparse tensor discriminant color space for face verification. Neural Networks and Learning Systems, IEEE Transactions on 23: 876–888. 10.1109/TNNLS.2012.2191620 24806760

[39] Wang S-J, Sun M-F, Chen Y-H, Pang E-P, Zhou C-G (2012) STPCA: sparse tensor principal component analysis for feature extraction. Pattern Recognition (ICPR), 2012 21st International Conference on. Tsukuba Japan. 2278–2281.

[40] Comon P (2002) Tensor decompositions. Mathematics in Signal Processing V: 1–24.

[41] Kolda TG, Bader BW (2009) Tensor decompositions and applications. SIAM review 51: 455–500.

[42] Candes EJ, Romberg JK, Tao T (2006) Stable signal recovery from incomplete and inaccurate measurements. Communications on Pure and Applied Mathematics 59: 1207–1223.

[43] Chartrand R (2007) Exact Reconstruction of Sparse Signals via Nonconvex Minimization. Signal Processing Letters, IEEE 14: 707–710.

[44] Chartrand R, Staneva V (2008) Restricted isometry properties and nonconvex compressive sensing. Inverse Problems 24 035020 doi:10.1088/0266-5611/24/3/035020

[45] Fischer A, Breuer F, Blaimer M, Seiberlich N, Jakob PM (2008) Introduction of a nonconvex compressed sensing algorithm for MR imaging. Proceeding of the 16^th^ Annual Meeting of ISMRM. Toronto Canada.

[46] Saab R, Chartrand R, Yilmaz O (2008) Stable sparse approximations via nonconvex optimization. Acoustics, Speech and Signal Processing, 2008. ICASSP 2008. IEEE International Conference on. Las Vegas USA. 3885–3888.

[47] Chartrand R (2009) Fast algorithms for nonconvex compressive sensing: MRI reconstruction from very few data. Biomedical Imaging: From Nano to Macro, 2009. ISBI ‘09. IEEE International Symposium on. Boston USA. 262–265.

[48] Majumdar A, Ward RK (2011) An algorithm for sparse MRI reconstruction by Schatten p-norm minimization. Magnetic Resonance Imaging 29: 408–417. 20952139 10.1016/j.mri.2010.09.001

[49] Andreopoulos A, Tsotsos JK (2008) Efficient and generalizable statistical models of shape and appearance for analysis of cardiac MRI. Medical Image Analysis 12: 335–357. 18313974 10.1016/j.media.2007.12.003

[50] Chartrand R, Wotao Y (2008) Iteratively reweighted algorithms for compressive sensing. Acoustics, Speech and Signal Processing, 2008. ICASSP 2008. IEEE International Conference on. Las Vegas USA. 3869–3872.

[51] Trzasko J, Haider C, Manduca A (2009) Practical nonconvex compressive sensing reconstruction of highly-accelerated 3D parallel MR angiograms. Biomedical Imaging: From Nano to Macro, 2009. ISBI ‘09. IEEE International Symposium on. Boston USA. 274–277.

[52] Majumdar A, Ward RK (2010) Under-determined non-cartesian MR reconstruction with non-convex sparsity promoting analysis prior. Medical Image Computing and Computer-Assisted Intervention–MICCAI 2010: Springer. 513–520. 10.1007/978-3-642-15711-0_64 20879439

[53] Ramirez-Giraldo J, Trzasko J, Leng S, Yu L, Manduca A, et al (2011) Nonconvex prior image constrained compressed sensing (NCPICCS): Theory and simulations on perfusion CT. Medical Physics 38: 2157. 21626949 10.1118/1.3560878 PMC3081867

[54] Trzasko JD, Haider CR, Borisch EA, Campeau NG, Glockner JF, et al (2011) Sparse-CAPR: Highly accelerated 4D CE-MRA with parallel imaging and nonconvex compressive sensing. Magnetic Resonance in Medicine 66: 1019–1032. 21608028 10.1002/mrm.22892 PMC3331793

[55] Majumdar A, Ward RK, Aboulnasr T (2012) Non-convex algorithm for sparse and low-rank recovery: Application to dynamic MRI reconstruction. Magnetic resonance imaging 31: 448–455. 23102947 10.1016/j.mri.2012.08.011

[56] Shiqian M, Wotao Y, Yin Z, Chakraborty A (2008) An efficient algorithm for compressed MR imaging using total variation and wavelets. Computer Vision and Pattern Recognition, 2008. CVPR 2008. IEEE Conference on. Anchorage USA. 1–8.

[57] Osher S, Burger M, Goldfarb D, Xu J, Yin W (2005) An Iterative Regularization Method for Total Variation-Based Image Restoration. Multiscale Modeling & Simulation 4: 460–489.

[58] Hitchcock FL (1927) Multiple invariants and generalized rank of a p-way matrix or tensor. Journal of Mathematics and Physics 7: 39–79.

[59] Hitchcock FL (1927) The expression of a tensor or a polyadic as a sum of products. Journal of Mathematics and Physics 6: 164–189.

[60] ElShahaby FEA, Landman BA, Prince JL (2011) Effect of regularization parameter and scan time on crossing fibers with constrained compressed sensing. Proceedings-Society of Photo-Optical Instrumentation Engineers: Image Processing. 79624J. doi:10.1117/12.878382. 10.1117/12.878382 PMC3087384 21552469

[61] Miao J, Huang F, Wilson D (2011) Investigation on Compressed Sensing Regularization Parameter using Case-PDM. Proceeding of the 19^th^ Annual Meeting of ISMRM. Montréal, Canada.

